# Monthly Summary

**Published:** 1873-10

**Authors:** 


*The Process of Taking Cold.*—Daily experience teaches the medical practitioner that persons who guard most anxiously against every possible chance of taking cold are most frequently its victims. Geiger, in an article on the mortality of children at Würtzburg, Germany, translated by Ch. Rauschenberg, M.D., Atlanta, Ga., shows that diseases of the respiratory organs cause, in the first year of life, the death of relatively many more legitimate than illegitimate children; while the contrary is true of diseases of nutrition, proving that the too great care of fond mothers to their offspring frequently produces what it is intended to prevent.—*Med. Record.*

